# Perspective: Improving Neonatal Registered Dietitian Nutritionist Staffing, Utilization, and Compensation

**DOI:** 10.1016/j.advnut.2025.100417

**Published:** 2025-03-24

**Authors:** Stephanie Merlino Barr, Tanis R Fenton, Rosa K Hand, Daniel T Robinson, Jae H Kim, Sharon Groh-Wargo

**Affiliations:** 1 Department of Pediatrics, MetroHealth Medical Center, Cleveland, OH, United States; 2 Cumming School of Medicine, University of Calgary, Calgary, Alberta, Canada; 3 Department of Nutrition, Case Western Reserve University, Cleveland OH, United States; 4 Division of Neonatology, Lurie Children’s Hospital and Prentice Women’s Hospital, Chicago, IL, United States; 5 Division of Neonatology, Cincinnati Children’s Hospital, Cincinnati, OH, United States

**Keywords:** registered dietitian nutritionist, staffing, compensation, retention, neonatal intensive care unit, organizational and workforce issues

## Abstract

Neonatal registered dietitian nutritionists (RDNs) are vital members of the multidisciplinary neonatal intensive care unit team due to their professional nutrition expertise and the critical role of nutrition for high-risk infants. The neonatal RDN is the only health care team member who is continually focused on infants’ nutrition status and nutrition care. They advocate for nutrition care at medical rounds and effectively improve nutrition and growth rates of critically ill infants, which helps to reduce health care costs. The purpose of this article is to describe how inadequate staffing, utilization, and compensation are contributing to neonatal RDNs leaving their clinical roles and to suggest solutions to the identified issues. Dedicated neonatal RDNs are recommended by the American Academy of Pediatrics; additionally, increased staffing of neonatal RDNs is desired within the profession to support best practices and to fill gaps with anticipated neonatal provider shortages. Research into ideal neonatal RDN staffing ratios to support improved patient care, professional development, and hospital cost savings are recommended. Utilization of neonatal RDNs at their full scope of practice can be achieved with increased staffing dedicated solely to infant/pediatric services and not in combination with adult services. RDN responsibilities, including ordering parenteral and enteral nutrition and managing infant nutrition preparation areas, improve patient care and provides opportunities for career advancement for neonatal RDNs. With the increasing costs for professional entry, adequate compensation for neonatal RDNs will likely be required to continue to attract and retain skilled practitioners in the field. Incorporation of RDNs in collective bargaining efforts, creation of career ladders, and establishment of billable services are strategies that could improve compensation. These changes should be solved by the collective efforts of dietitians, neonatologists, clinical nutrition managers, and hospital administration.

## What is the Problem?

Critically ill newborn infants born prematurely or with congenital anomalies require collaborative intensive care. Despite declining birth rates, the number and complexity of neonatal intensive care unit (NICU) admissions continue to increase; nutrition has grown to be a central treatment for these high-risk infants to thrive and survive [1]. Parenteral nutrition, human milk, specialized enteral products, and micronutrient supplementation are vitally important therapies. Although the entire NICU team considers nutrition care, the neonatal registered dietitian nutritionist (RDN) has nutrition as their sole focus and priority.

Neonatal RDNs are vital members of the NICU team due to their professional nutrition expertise and the critical role of nutrition in the care of high-risk infants. Neonatal RDNs’ importance is frequently emphasized by physicians and leaders in neonatology. As RDNs and physicians with wide professional networks, we are aware of many neonatal RDNs leaving the clinical field, or the profession entirely, due to issues that could be improved via systemic change.

The major objective of this *Perspective* article is to respond to what was learned from a survey of neonatal RDN practice, which highlighted a need for increased neonatal RDN staffing [[2], [3], [4]]. Our survey findings have sparked numerous conversations regarding opportunities and concerns of the neonatal RDN profession [[2], [3], [4]]. Our survey identified 3 areas of concern: staffing, utilization, and compensation. We aim to describe these problems, highlight perspectives of experts in the field, and propose actions to better support these vital professionals.

## Increased Neonatal RDN Staffing Is Needed

“Caring for premature infants born before 24 weeks’ gestation is critically dependent upon a multidisciplinary team in which the foundation for success is built upon the complex importance of nutritional support from birth to discharge and beyond. Growth of both the body and the brain must be supported… to optimize neurological intact survival. The best way to achieve nutritional excellence for this incredibly fragile patient population is to incorporate a dedicated neonatal registered dietitian as one of the foundational keystones of your tiny-infant team.”

*Jonathan M. Klein, MD*

*Division of Neonatology, University of Iowa Stead Family Children’s Hospital*

*Professor Emeritus, University of Iowa*

“In practice, I have always depended on the expertise of registered dietitian nutritionists. Both the recent Standards for Levels of Neonatal Care II, III and IV (Pediatrics, 2023∗) and Guidelines for Perinatal Care, 8th edition (American Academy of Pediatrics, American College of Obstetrics and Gynecology, 2017) suggest at least one [RDN] should be available to serve only the NICU [5]. I think a real issue is that there aren’t enough neonatal dietitians, which explains why we used the phrase ‘at least one.’ Obviously, NICUs with high volume would benefit from more.”

*Ann Stark, MD*

*Director of Faculty Development, Department of Neonatology, Beth Israel Deaconess Medical Center*

*Professor in Residence of Pediatrics, Harvard Medical School*

The neonatal RDN is the only health care team member who is continually focused on infants’ nutrition status and nutrition care [2]. They advocate for nutrition care at medical rounds and effectively improve nutrition and growth rates of critically ill and unstable high-risk infants, which helps to reduce health care costs [[6], [7], [8], [9]]. They are necessary for optimizing specialized nutrition support for infants with metabolic abnormalities, identifying and treating those with acquired nutrient deficiencies, and preventing malnutrition [10].

Although there is not an identified staffing ratio of neonatal RDNs for the promotion of ideal patient outcomes, an increase in current neonatal RDN staffing is desired within the profession to support best practices [2]. The American Academy of Pediatrics recently recognized the neonatal RDN as essential members of the NICU team, with recommended staffing minimums based on NICU level [5]. The presence of a neonatal RDN in the NICU supports improved rankings for United States’ pediatric hospitals [11]. Recent staffing surveys in Canada, Australia, and New Zealand have estimated that the majority of level III/IV NICUs have access to neonatal dietitian services [3,10]. In the United States, country-wide data on RDN staffing are lacking, but many NICUs, including level III and IV units do not have routine RDN coverage. Despite the importance of neonatal RDNs, inadequate staffing in some NICUs, and the desire for more of these professionals in the NICU setting, this author group is concerned that neonatal RDNs are leaving clinical dietetics or the profession entirely for better opportunities for professional growth and compensation.

The number of credentialed RDNs in the United States has been relatively consistent at just over 111,000, with ∼4000 newly credentialed RDNs entering the field annually [12,13]. It is unknown whether this workforce is adequate to fill all clinical RDN positions, as the last comprehensive study of workforce capacity and demand occurred in 2012, well before the workforce disruptions caused by the COVID-19 pandemic [14]. The Commission on Dietetic Registration is undertaking a new workforce capacity and demand study to answer these questions and to evaluate whether the pipeline of RDNs is adequate [15]. There have been reductions in student enrollment in undergraduate dietetics programs (Didactic Program in Dietetics) and dietetic internships [16]. Some of this downward trend has been offset by increases in students enrolling in Future Education Model Graduate Programs, leading to a net consistent number of new examination candidates [16]. These new program types help late comers enter the dietetics field more efficiently than the traditional Didactic Program in Dietetics to dietetic internship route, which may help increase RDN numbers over time. Although NICU RDNs are highly experienced and educated as a group, there are decreasing training opportunities for those looking to enter neonatal nutrition [2]. Support of pathways to become specialized in neonatal nutrition is necessary to ensure the sufficient availability for a workforce to staff NICUs.

## Neonatal RDNs May Help Address Staffing Concerns in Pediatric Care

“The first days and weeks [of life] focused on nutrition and growth are essential to optimize long-term health outcomes… pediatric training is changing with decreasing [training] time allotted for critical care. This is exceptionally worrisome as future neonatologists receive less education in nutrition. Neonatal RDN expertise is crucial and needed now more than ever.”

*Daniel T. Robinson, MSc, MD*

*Attending Physician, Division of Neonatology, Lurie Children’s Hospital and Prentice Women’s Hospital*

*Associate Professor of Pediatrics, Northwestern University Feinberg School of Medicine*

Staffing concerns within the NICU environment are not unique to the RDN. Pediatric fellowship fill rates dropped to 77.5% in 2024 [17]. Recently, the Accreditation Council for Graduate Medical Education issued new program requirements for pediatric residencies reducing the minimum NICU rotations from 8 to 4 wk, to be enforced by 2025 [18]. Simultaneously, pediatric residency vacancies notably increased, with 8.2% of positions unfilled in the 2024 match, increasing from 1.4% to 2.9% vacancy rates seen from 2021 to 2023 [19]. Neonatal fellowship position vacancies have also increased from 2.8% to 11.3% in the same period [20]. There are concerns about sufficient medical trainees to continue to support the pipeline of neonatologists as well as a growing need and limited availability of advance practice providers to supplement the changes in physician workforce [[20], [21], [22]].

Shortened residency exposure and unfilled pediatric fellowship positions, particularly those in neonatal-perinatal medicine, positions will result in unmet clinical needs in the NICU, which may be partially filled by neonatal RDNs. Recognizing the potential of staffing shortages of providers in the NICU and other pediatric units, improving RDN staffing in this environment can support these changes in workflow while concurrently improving quality of treatment plans and nutritional care.

## Neonatal RDNs Should Be Used at Their Full Professional Scope

“Neonatal RDNs are critical in educating the entire care team on neonatal nutrition, ensuring adherence to protocols, tracking neonatal growth and nutritional outcomes—key drivers of overall health—and leading quality improvement and research initiatives to enhance nutritional delivery. A NICU without a neonatal RDN on the team misses a vital opportunity to provide the highest quality of care.”

*Camilia Martin, MD, MS*

*Division Chief of Neonatology, New York-Presbyterian Hospital*

*Professor of Pediatrics and Endowed Professor of Neonatology, Weill Cornell Medical College, Cornell University*

Training to become an RDN requires completion of an accredited undergraduate science degree, a supervised dietetic internship, as well as a master’s degree (requirement implemented January 2024) before sitting for the credentialing examination. The scope of RDN practice in the United States is defined by the Commission on Dietetic Registration Scope and Standards of Practice Task Force, state licensure boards, and national legislation [23]. The neonatal RDN’s scope of practice can include ordering parenteral and enteral nutrition, managing human milk and formula preparation areas, and leading quality improvement and research projects. These items of advanced practice are areas of high interest among neonatal RDNs, and evidence suggests that improvement in RDN staffing could support development of these professional responsibilities [2].

In 2014, the Centers for Medicare and Medicaid Services updated their regulations to include RDNs as qualified professionals to write therapeutic diet orders (including parenteral and enteral nutrition) as authorized by medical staff and in accordance with state law [24]. When nutrition orders are written by RDNs errors and hospital costs are lower and the quality of nutrition provided improves [[25], [26], [27]]. In the NICU, RDN order writing privileges can reduce medical provider workload and rounding time, important benefits in the setting of potential provider shortages.

Recent infant nutrition product recalls and shortages of enteral and parenteral nutrition products have highlighted the complexity of infant nutrition and the importance of the RDN in navigating this expansive field. Neonatal and pediatric RDNs demonstrated the need for their expertise in disaster planning teams, coordination with supply chain, communicating with industry representatives, and translation of United States Food and Drug Administration regulations while rapidly updating nutrition plans in inpatient and outpatient settings and ensuring patient safety. Inclusion of these uniquely qualified nutrition experts on administrative teams is necessary to ensure quality and safe patient care.

Neonatal RDN management of infant feeding preparation spaces similarly allow for advancement in RDN practice and quality of patient care. Preparation of infant feeds away from the bedside in a dedicated space is considered a standard of care in the NICU environment [5]. Centralized milk handling reduces human milk misadministration, feeding expired human milk, preparation errors, and microbial contamination of infant feeds [[28], [29], [30]].

Supporting neonatal RDNs’ performing at full professional scope allows for career advancement that is often lacking in clinical dietetics, which may be a contributor to professionals leaving the field. Support of neonatal RDN advancement should be considered a necessity for both health care professionals as well improvements in patient care. However, these advancements must be paired with appropriate staffing and compensation.

Supporting neonatal RDNs practicing at their professional scope should be supported by including nutrition and dietetics technicians, registered (NDTR) and milk room technicians in the NICU workforce. NDTRs are associate’s degree-prepared professionals who can screen admitted patients for nutritional risk, lead infant feeding preparation, and perform anthropometric measurements, among other important clinical tasks in the NICU. Although current staffing practices of NDTRs in United States NICUs are not well understood, their employment is a potential avenue to improve staffing and support neonatal RDNs in expanding their clinical practice [2], thus supporting patient care and the entire NICU team.

## Neonatal RDNs’ Expertise Warrants Improved Compensation

“The role of the neonatal dietitian has become increasingly vital in the NICU because of the rising complexity of nutrient delivery to vulnerable newborns. It is imperative that neonatal dietitians are required for each NICU, supported in effort and appropriately compensated for their unique expertise in optimally caring for NICU infants. Their daily decisions are depended upon by the medical providers and directly impact the growth of these infants that, in turn, ultimately affect the quality of their long-term health and developmental outcomes.”

*Jae Kim, MD, PhD*

*Director, Division of Neonatology and Co-Director, Perinatal Institute, Cincinnati Children’s Hospital Medical Center*

*Professor of Pediatrics, University of Cincinnati*

**FIGURE 1:**
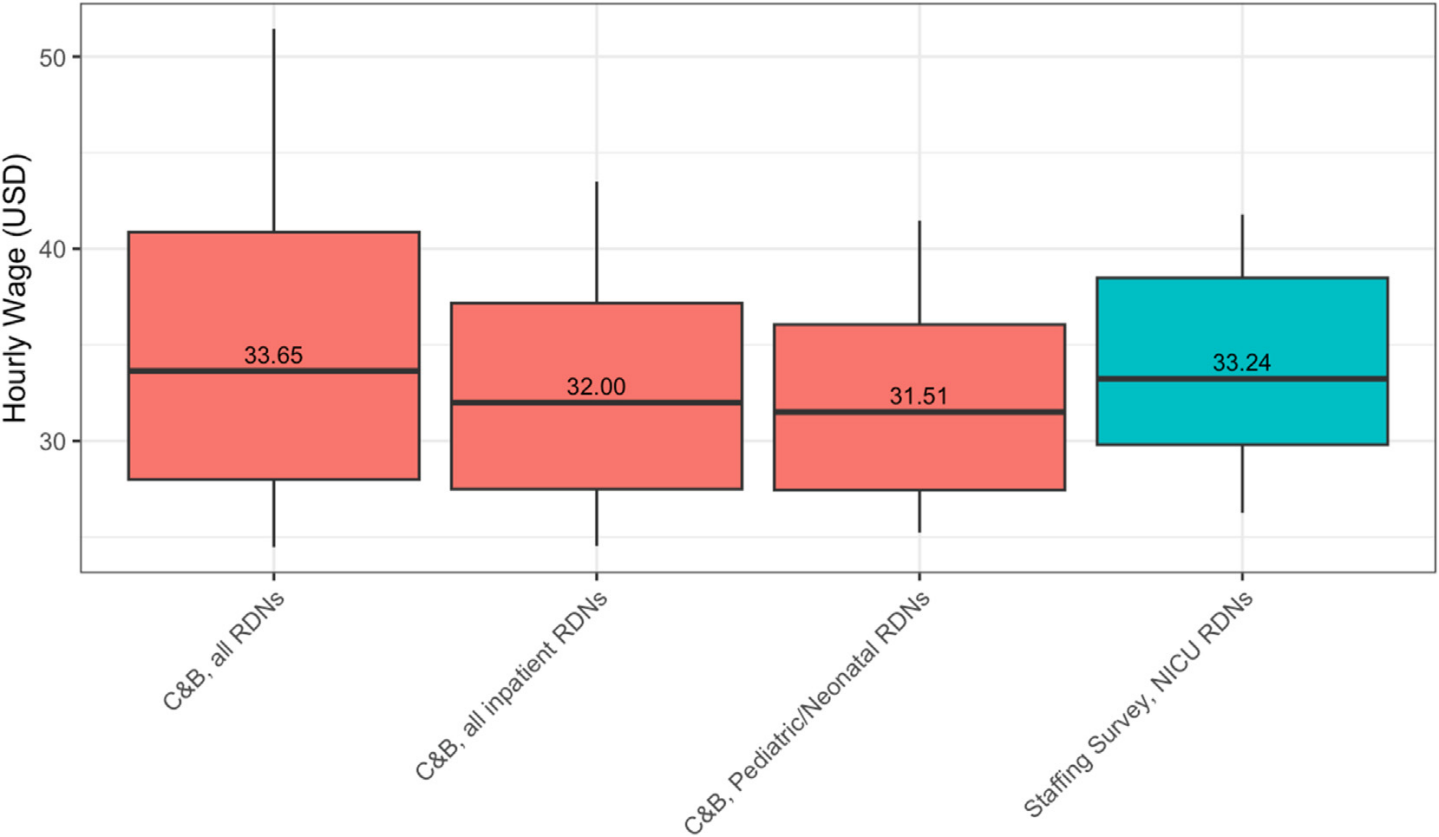
Hourly wages of registered dietitian nutritionists in the United States, 2021. Data sources: Academy of Nutrition and Dietetics Compensation and Benefits Survey of the Dietetics Profession [4]. C&B, Academy of Nutrition and Dietetics Compensation and Benefits Survey of the Dietetics Profession; NICU, neonatal intensive care unit; RDN, registered dietitian nutritionist; USD, United States dollar.

Neonatal RDNs’ training and expertise merits appropriate compensation and opportunities for meaningful advancement within clinical dietetics. In 2021, United States neonatal RDNs reported a median hourly wage of $33.24 (interquartile range, $29.81–$38.49) [2]. When compared with other clinical dietitians, neonatal RDN’s compensation was not meaningfully different—despite having specialized training and education (Figure 1) [31]. Neonatal RDNs in the United States also make comparatively less than neonatal RDNs in Canada [3].

**FIGURE 2:**
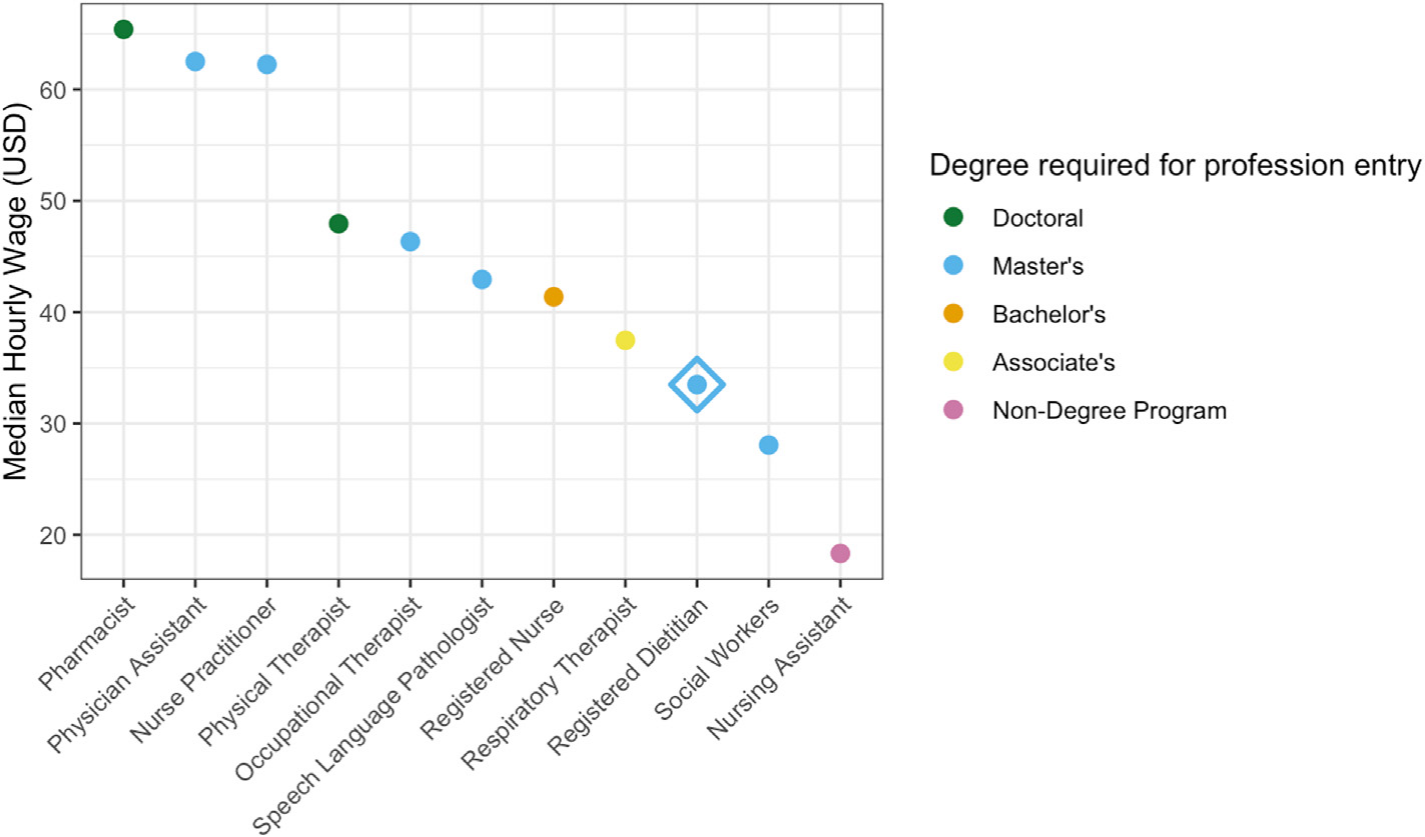
Median hourly wage of health care professionals. Data from United States Department of Labor Bureau of Labor Statistics, 2023 (https://www.bls.gov/ooh/). Physicians excluded due to wide salary range of profession not reflective of typical neonatologist wage. These data are reflective of registered dietitian nutritionist (RDN) wage before master’s degree requirement for profession entry, implemented January 2024. However, at a similar period to the wage data collection, 57% of practicing RDNs had a master’s degree and 4% held doctoral degrees, thus we present RDNs as being a master’s degree-prepared profession [32]. USD, United States dollar.

Despite their high educational and training requirements, neonatal RDNs are among the lowest compensated members of the NICU team (Figure 2) [32]. We suggest that compensation of the neonatal RDN should be similar to those of other allied health professionals in the NICU. The median salaries of speech language pathologists are 28% larger than registered dietitians in the United States, occupational therapists earn 38% more, and physical therapists earn 48% more [33]. This discrepancy in compensation is despite neonatal RDNs filling a similar type of role with their own unique expertise and requiring a similar level of training.

Adequate compensation for neonatal RDNs will likely be required to continue to attract skilled practitioners into the field, especially given the increasing costs for professional entry. Becoming a registered dietitian now requires a 4-y undergraduate degree, a master’s degree, and a dietetic internship, which is typically unpaid. Improved compensation also recognizes the advanced skills and knowledge of graduate degree holding RDNs.

## Calls to Action

We suggest the following to improve staffing of neonatal RDNs:

- Physician and hospital leaders advocate for increasing dietitian staffing in the NICU environment.
- Neonatal RDNs have protected NICU time that is commensurate with the many clinical, administrative, and educational duties required to support excellence in care and patient safety. Employment of neonatal RDNs within neonatology is an approach to ensure this.
- Further research into ideal neonatal RDN staffing ratios to support improved patient care and professional development is needed.
- Evaluate staffing ratios to support hospital cost savings in the setting of anticipated neonatology and pediatric provider shortages.
- Increase neonatal RDN staffing levels by 30%, as desired by neonatal RDNs [2,3].

We suggest the following to support maximizing utilization of neonatal RDNs at their full professional scope:

- Managers assist in the evaluation of job descriptions and creation of career ladders to promote growth within clinical roles.
- NICUs employ RDNs for milk laboratory/formula room management.
- Hospitals create policies that support neonatal RDN parenteral and enteral order writing privileges in accordance with state laws.

We suggest the following to promote the improvement in compensation of neonatal RDNs:

- Neonatal RDN compensation is increased to reflect the level of experience as well as training and education of the practitioner.
- Compensation of neonatal RDNs is increased to be comparable with other allied health professionals that fill similar roles in the NICU team.
- RDNs use collective bargaining efforts and participate in multidisciplinary discussions to support improved staffing and compensation.
- Establish billable services of neonatal RDNs and separate their work from the hospital bed charge.
- Promote legislation to support reimbursement for dietitian evaluation and nutrition intervention.

Neonatal RDNs have been contributing to the multidisciplinary NICU team for over 50 years, and their role is supported and valued by their physician colleagues [34]. NICU patients continue to increase in number and medical complexity and strains throughout the health care system continue to develop. Empowering and supporting the neonatal RDN is a way to improve ongoing quality care and patient successful outcomes. This *Perspective* is a call to action for dietitians, neonatologists, clinical nutrition managers, and hospital administration to enact change—we have work to do!

## Author contributions

The authors’ responsibilities were as follows – SMB, TRF, RKH, SG-W: conceptualized and wrote the manuscript; and all authors: contributed to the revisions and have read and approved the final manuscript.

## Funding

The authors reported no funding received for this study.

## Conflict of interest

SG-W reports speaking and lecture fees from Abbott Nutrition. SMB reports speaking and lecture fees from Abbott Nutrition and Mead Johnson Nutrition. JK reports consulting or advisory for Medela, IBT, NEC Society, Carag, Biomilq, and Cardinal Health and equity or stocks in Astarte Medical and Nicolette. DTR reports consulting or advisory and speaking and lecture fees from Baxter. All other authors report no conflicts of interest.
